# A qualitative study on blood and marrow transplant recipients’ perceptions of health professional roles following BMT and preferences for ongoing care

**DOI:** 10.1007/s11764-024-01658-4

**Published:** 2024-08-17

**Authors:** Gemma McErlean, Christine Ashley, Anisha Pradhan, Vanessa Yenson, Alana Paterson, Gai Farnham, Fran Owen, Anne-Marie Watson, Peter Presgrave, Ian Kerridge, Elizabeth Halcomb

**Affiliations:** 1 School of Nursing, Faculty of Science, Medicine & Health, University of Wollongong, Northfields Ave, Wollongong, NSW Australia; 2 St George Hospital, Kogarah, NSW Australia; 3 Consumer Advocate, Faculty of Health, University of Technology Sydney, Ultimo, NSW Australia; 4 Liverpool Hospital, Liverpool, NSW Australia; 5 Wollongong Hospital, Wollongong, NSW Australia; 6 Royal North Shore Hospital, St Leonards, NSW Australia Sydney,

**Keywords:** BMT, Allogeneic transplantation, Autologous transplantation, Long-term care, Shared care, Survivor preference, Survivorship

## Abstract

**Purpose:**

Survivors of blood and marrow transplantation (BMT) require life-long follow-up involving both tertiary transplant and primary care services. This paper explores the attitudes and preferences of BMT survivors and their carers regarding the transition from BMT centre care to primary care*.*

**Methods:**

This qualitative study involved semi-structured interviews with BMT survivors and carers from New South Wales, Australia. Interviews were audio-recorded, transcribed verbatim and thematically analysed.

**Results:**

Twenty-two BMT survivors and six carers were interviewed. Two themes emerged: (1) ‘Relationships with health professionals’ and (2) ‘Challenges of long-term care’. Participants, particularly rural/regional survivors, had diverse views on the availability of community BMT expertise and identified a range of strategies to optimise care for BMT survivors.

**Conclusions:**

These results highlight the importance BMT survivors and carers place on their relationships with, and ongoing access to, specialised BMT teams for long-term care. While some are happy to receive community-based care, concerns exist about the capacity of primary care providers, particularly in rural and regional areas. Improved support, communication and coordination between BMT centres and primary care may help facilitate a person-centred, sustainable shared care model. Provider education, use of telehealth and clear delineation of roles and responsibilities may assist in this transition.

**Implications for Cancer Survivors:**

As BMT survivors live longer post-treatment, transitions of care and sustainable long-term care models are needed. A shared care approach, integrating specialised BMT teams and local primary care, may optimise outcomes but requires further development to balance accessibility, preferences, and specialised care needs.

## Introduction

Blood and marrow transplantation (BMT) is a well-established treatment for many malignant haematological, immunological and metabolic conditions and may provide the only possibility for long-term survival in some patients [1]. As the number of transplants and the rates of survival following BMT have improved [2], increasing numbers of patients are surviving long-term. In 2019, almost 50,000 BMTs were reported [3], with 10-year survival rates of up to 97% depending on the primary disease, age at transplant and transplant type [4]. However, both allogeneic and autologous BMT are associated with significant morbidity and mortality. This includes many serious long-term and late sequelae such as secondary malignancy, graft versus host disease, infection, infertility, metabolic syndrome and other chronic conditions [5, 6]. As these late effects can impact life expectancy and quality of life, BMT survivors must have access to long-term follow-up following transplantation [7, 8].

Due to the diversity of potential sequelae experienced by BMT survivors, they may require up to 34 assessments or consultations each year, involving some six different clinical specialties [1]. While there is broad agreement about the importance of this follow-up, the increasing number of survivors makes the demands related to such follow-up on existing BMT centres unsustainable. At the same time, many BMT centres are experiencing limitations in staff, data management and resources [8]. Consequently, current long-term follow-up programmes are of variable quality and may provide care that is inconsistent with international and national best practice guidelines [7, 8].

To address these challenges, new models for long-term care are being explored. These include establishing satellite centres with local or visiting haematologists with local monitoring and assessments in primary care settings [1, 9, 10]. Models where care is shared between specialist and primary care services are already used in chronic disease and cancer management; it has successfully reduced the burden on specialists [11]. In several cases, these shared care models used telehealth and videoconferencing to improve communication between patients, local healthcare providers and specialists [11–13].

It is recognised that BMT survivors and their carers experience significant levels of stress before, during and after BMTs that impact their well-being and experiences of care [14, 15]. Like patients with other cancers and chronic conditions [16, 17], BMT survivors have been reported to appreciate continuity of care and the development of trust with their key care providers [18]. There has, however, been limited research exploring survivors’ preferred models of long-term follow-up care or their perceptions of health professional roles in monitoring their well-being [10]. Understanding patient and carer perspectives is important in ensuring that new models of care meet their needs. Therefore, this study sought to explore BMT survivors’ and their carers’ experiences of long-term care.

### Purpose

This paper seeks to describe the attitude and preferences of BMT survivors and their carers regarding the transition of care from the BMT centre to primary care.

## Methods

A qualitative descriptive approach guided in-depth, semi-structured interviews with BMT survivors and carers. [19]. The Consolidated Criteria for Reporting Qualitative Studies (COREQ) [20] checklist was used to guide reporting.

BMT survivors were eligible to participate if they were; aged 18 years or older, in remission; more than one year post-BMT; able to read and speak English; and not experiencing a life-limiting condition. BMT clinicians identified potential participants from databases of metropolitan BMT centres in two health districts in NSW, Australia. Potential participants were contacted, provided study information and invited to participate by the clinicians. A list of consenting participants was provided to the research team, who then contacted each person and scheduled an interview at a mutually convenient time via ZOOM videoconferencing. Survivor participants were also asked if they had a carer who could be invited to participate. The nominated carer was sent study information and invited to participate in a separate interview.

### Data collection and analysis

A semi-structured interview schedule was developed based on the literature and expert input. Initially, demographic data were collected, followed by questions exploring participants’ perceptions and preferences regarding long-term care. This included items about their understanding of long-term care post-BMT, the role of health professionals in their long-term care and their experiences and thoughts about shared care. Questions were pilot-tested to check comprehensiveness and comprehensibility, and minor adjustments were made to improve clarity. All interviews were undertaken by five registered nurses with significant experience working with BMT and cancer patients. Participants were given pseudonyms and interviews were audio-recorded and transcribed by a professional transcription service.

Transcripts were uploaded to NVivo Version 14™ (QSR International Pty Ltd, 2012) and checked for accuracy by one researcher. Braun and Clarke’s [21] six-step approach to thematic analysis was used. This involved researchers (GM, CA, AP, EH) (1) becoming familiar with the data, (2) generating initial codes, (3) refining codes and developing themes, (4) using consensus to further refine and revise themes, (5) naming and defining themes, and (6) detailing the findings in this report.

### Ethics

The Human Research Ethics Committees of the South Western Sydney Local Health District (Approval No. 2022/ETH01503) and the University of Wollongong approved this project. All participants voluntarily agreed to participate and provided written consent.

## Results

Twenty-two BMT recipients participated, including 18 males (81.8%) and four (18.2%) females (Table 1). All participants were greater than 2 years post-BMT, with six (22.3%) at five or more years post-transplant. Fifty-four percent (*n* = 12) of participants were from metropolitan locations, 31.8% (*n* = 7) lived in regional areas and 13.6% (*n* = 3) were from rural locations. All survivors were receiving long-term post-BMT follow-up care through their transplant centre including cancer screening, vaccination, prevention and treatment of long-term and late effects of BMT and chronic co-morbidity, health promotion and disease prevention. Six carer interviews were also completed.

**Table 1:** Participant demographics

	Pseudonym	Age	Transplant type	Disease	Gender	Location	BMT (year)
BMT survivors	Alex	58	Autologous	Multiple myeloma	Male	Regional	2021
	Belinda	59	Autologous	Multiple myeloma	Female	Regional	2020
	Chris	30	Allogeneic	Acute leukaemia	Male	Metropolitan	2020
	Daniel	64	Autologous	Multiple myeloma	Male	Regional	2019
	Ethan	55	Autologous + Allogeneic	Lymphoma	Male	Metropolitan	2014 + 2015
	Fred	50	Allogeneic	Acute leukaemia	Male	Metropolitan	2016
	Gabriel	71	Autologous	Multiple myeloma	Male	Metropolitan	2020
	Henry	64	Autologous	Multiple myeloma	Male	Metropolitan	2019
	Isabella	54	Allogeneic	Acute leukaemia	Female	Regional	2021
	John	51	Autologous	Lymphoma	Male	Regional	2019
	Kevin	50	Autologous	Lymphoma	Male	Metropolitan	2021
	Liam	67	Autologous	Multiple myeloma	Male	Metropolitan	2019
	Margaret	40	Autologous	Multiple myeloma	Female	Metropolitan	2021
	Noah	80	Autologous	Multiple myeloma	Male	Metropolitan	2017
	Oliver	66	Autologous	Myelofibrosis	Male	Rural	2005
	Paul	67	Autologous	Lymphoma	Male	Metropolitan	2020
	Una	63	Autologous	Multiple myeloma	Female	Metropolitan	2021
	Roger	64	Allogeneic	Lymphoma	Male	Metropolitan	2021
	Steve	70	Allogeneic	Acute leukaemia	Male	Rural	2019
	Trevor	37	Allogeneic	Acute leukaemia	Male	Regional	2019
	Victor	67	Allogeneic	Aplastic anaemia	Male	Regional	2020
	Will	68	Autologous	Lymphoma	Male	Rural	2020
				Relationship			
Carers	Sophie	63	Allogeneic	Wife	Female	Rural	
	Campbelle	49	Autologous	Wife	Female	Metropolitan	
	Anthony	65	Autologous	Husband	Male	Regional	
	Emma	56	Allogeneic	Wife	Female	Rural	
	Clinton	65	Autologous	Husband	Male	Regional	
	Lily	40	Allogeneic	Daughter	Female	Metropolitan	

Two themes were drawn from the data: (1) 'Relationships with health professionals’ and (2) ‘Challenges to long-term care’.

### Theme 1 Relationships with health professionals

Participants described the value and nature of the relationships they developed with the BMT team and the varying roles played by the team and their general practitioners (GPs) during and after the BMT. For some, the level and quality of care were overwhelming:

It’s very humbling this process where you’re a fellow that’s finished paying his tax for life now and they still spend a million dollars on you to keep you alive. It’s a wonderful system (Trevor - BMT survivor).

I got better [laughter]! The friendships I made with A and G and Dr. D …. I can’t forget [crying] (Oliver - BMT survivor).

Three sub-themes highlighted relationships with the haematologist, transplant co-ordinators and GPs.

#### Haematologists

Several participants spoke of the strong bond they felt towards the haematologist who had guided them through the BMT process:

Dr [Haematologist], he’s been outstanding, absolutely. Not only is he great at what he does, he’s just a wonderful fellow (Ethan - BMT survivor).

I think the specialists there are fantastic. Yeah, I think they are cutting edge, they really know what they’re doing (Henry - BMT survivor).

I just – someone prolongs your life, or does what they do, you just – I don’t know, it’s a very special bond you end up having with them. More than you get with your GP or – you know what I mean? … these people have your life in their hands more than most doctors (Alex - BMT survivor).

For some participants, this bond was evidenced by two-way respect and understanding:

My haematologist and I... I’m quite open to talking and that, and she’s pretty blunt... we sort of work out what’s going on between us. She runs the show, but I’ll say to her – like I was on higher [medication], and I just said to her, “I reckon we could drop down.” And we dropped down and my numbers didn’t change, and she sort of goes, “Yes, good call” (Alex - BMT survivor).

Some survivors and carers described the profound trust they had in their transplant physician and their belief that the failure of other health professionals to communicate with and defer to their BMT team/physician compromised their care and created further risks of serious morbidity and mortality.

And there’s a gap I find between the hospital staff recognising what he’s [BMT survivor] had, and that he’s had his spleen removed, and he’s had a transplant, and that’s the reason he’s sick, where they just want to treat him for the sickness. And we have to say to the doctors, “Can you please call Doctor [BMT doctor] and tell him that [BMT survivor] is here, because it’s all connected.” And the last time he was in with RSV, I was there, and I said to the doctor, “Are you going to tell Doctor 1[BMT doctor]?” and he goes “Oh, well, I wasn’t going to, because it’s a chest and lung problem.” And I was like, “No, no, can you please go and tell his doctor, because he needs to know that he’s unwell” (Lily - carer).

#### Transplant co-ordinators

Most participants described the important role of transplant co-ordinators in providing physical, psychological and moral support during the period of hospitalisation for transplant:

The nurses and everything were just out of control good; they were – plus you’re awake so much for a lot of the time, they do any – you know, some of them work double shifts straight. You should see how much time they put in. And they were just awesome... (Alex - BMT survivor).

During my hospital stay I think I only saw two visitors, in 30 days, so I relied on the nursing staff…you know what I mean? They were lovely (Alex - BMT survivor).

Participants described how the role of transplant co-ordinators and their importance to BMT survivors changed following their discharge home, with BMT co-ordinators becoming more a source of advice and support, rather than a provider of direct care. For many participants, this shift in the roles and responsibility of transplant co-ordinators contributed greatly to their confidence in their ability to self-manage their health and their trust that a successful transition was possible:

She [BMT co-ordinator] was really, really good. I’d ring her up if I needed to know something or whatever. I would have gone to her a few times, you know. Because I didn’t know what I was supposed to be doing even though it says it all in the brochure (Una - BMT survivor).

Any concerns I had and there [were] numerous ones that I can recall. But I would just ring [BMT co-ordinator] and tell her what was going on and she would talk to [BMT Doctor] and they would get back to me. [BMT co-ordinator] was always available and if I left a message, she always rang back. She was absolutely fantastic, so having that reassurance that you have the follow-up was great (Steve - BMT survivor).

The [BMT co-ordinator] made me feel so comfortable that you can ring them any time. Like I wouldn’t feel uncomfortable ringing [BMT co-ordinator] now if I had a concern. She would be my first line of contact before my GP (Steve - BMT survivor).

Some participants suggested that post-BMT nurse-led models of care could provide an alternative to reliance upon local GP services, particularly where patients experience difficulty accessing primary care services in the community:

…she [BMT co-ordinator] rang me to make sure I was eating, making sure I was OK, if I had any questions. So in that respect, that nurse did a lot for me… Definitely a nurse (Una - BMT survivor).

…it probably needs to be something like a daily home nurse visit to do blood pressure, oxygen levels, and heart rates and the like. I don’t know whether it still exists; when I first had the heart episodes, there was a thing working out of XXXX Hospital called the Tach Team. Where you’d have a Tach Team nurse would come around daily to check to see that your heart was in rhythm and blood pressure and the like. So that’s something that they could probably look at for stem cell post…But as I said previously, I think some form of a nurse Tach Team type situation daily would probably be more efficient in the first term. And then if they said you need to either contact to the GP or the haematology specialist, that could be then probably more immediately arranged by a Tach Team nurse than the patient doing it themselves...(Daniel - BMT survivor)

#### General practitioners

Participants’ views of the role of the GP during the BMT journey varied. Several participants spoke of not having continuity of care with a specific GP, which impeded their experience. Alex explained: *It’s not like the old days when you had a really strong affiliation with your doctor. You don’t always get the same doctor, doctors come and go in country towns.* Henry also described that *a lot of people just don’t have a regular GP, because with GPs it’s very rare for a GP to actually be in the one spot for a long time*. While Henry currently had a GP that he trusted who *sort of keeps on top of what I’ve got…. if she moved or retired or something like that I don’t know. Yeah, it would be back to who knows what (Henry—BMT survivor)*.

Beyond having continuity of care with a GP, participants raised concerns about being able to easily access their GP:

Unfortunately down here… there’s not many GPs. ... It’s getting in to see these blokes (Victor - BMT survivor).

…the time it takes to get an appointment, phone appointment or a physical consult with your GP, is never instant, basically (Daniel - BMT survivor).

You’ve got to book about a month in advance to see him…(Liam - BMT survivor).

Other participants, however, reported more positive experiences with follow-up from their GP:

[the] local GP would slip me straight in because they knew I was a cancer case. And they gave me a little more, maybe little more priority over some … (Will - BMT survivor).

…my GP… And she is fantastic. And when I was going through all this last year, she would just say to her staff if she would get my ring [phone call], put it straight through, don’t ask much. She would just make time for me. And that was so good because, you know, if I needed medication or anything like that or whatever, she would take my call (Una - BMT survivor).

In addition, even when survivors had access to a regular GP, some carers raised issues of their loved one being reluctant to share information about their condition with their GPs, preferring to the haematologist with concerns. In the view of one carer, he felt his partner had made it difficult for the GP to provide effective follow-up and management of any health issues:

He doesn’t really follow it up, no. He’s really funny, even when he’ll go to the doctor for something. Like he was sick – I said, “Did you tell him about your medical history?” “They don’t need to know that, what do I need to tell them that for?” Yeah, that’s just him, he won’t say... Yeah, he’s not one to really talk about how he feels, he’ll bottle it up and not say anything (Campbelle - carer).

### Theme 2: Challenges to long-term care

Many participants highlighted a number of challenges associated with adhering to long-term care requirements.

- Distance/time burden

A major issue highlighted by participants was the burden associated with long-term specialised care at BMT centres. For those living in rural and regional areas, the cost, time commitment and inconvenience of regular visits to the BMT centres were particularly challenging. These challenges were made all the more difficult by the perception of BMT survivors who thought that their BMT team lacked sufficient understanding of the burden of long-term care and rarely expressed empathy with them.

The testing and the basic tests that I had to do, like my bone density test, my lung function tests, those sorts of things. I didn’t need to see the specialist for that... It’s not just an hour and forty-five minutes out of my day it’s an hour and forty-five minutes one way, it's an hour and forty-five minutes back and there’s petrol... parking… stopping for lunch... That’s a whole day’s worth of work that I’m missing… But I don’t live there [in Sydney], I don’t want to live there and that was an issue with the doctors... they couldn’t sort of understand why I wanted to get back to my place (Victor - BMT survivor).

In contrast, a number of participants described how provision of ‘low technology care’ locally where possible, significantly reduced the burden on patients and their cares:

I can go back to them but they’re about an hour and forty-five minutes from where I live. I did have to have a venesection…too much iron in my blood. So they had to drain a litre of blood every month for six months there. I was able to do that at [local] Hospital which is a lot closer to where I am (Victor - BMT survivor).

Some participants specifically identified the potential for technology to optimise the management of long-term care. A carer who struggled with remembering the multitude of appointments thought things could be improved by receiving text or email reminders from their haematologist to attend follow-up appointments with their GP. Others expressed how a flexible approach to communication with care providers had benefited them, such as being able to use telehealth instead of face-to-face consultations when things were going well.

You’ve got a patient’s information, mobile or email thing that you, you know you just trigger off the button and go, hey, it’s been flagged up in our system that you’re due to see your GP for this check-up. That would be perfect because people get busy in their life because they’re feeling better than what they did when they were prior to the treatment and through the treatment. So they just get on and forget about it (Campbelle - carer).

I found it much more compatible to have the option to either go there or do the video link. So because I was travelling so well, I was comfortable with the video link and it took the burden of the travel away. But it was nice to know that if anything was wrong, I could go there and talk face to face (Steve - BMT survivor).

Another carer suggested that providing online access to test results could help patients to take more responsibility for their progress:

A patient should be able to log on and see their results or his results. And I think the patient should be informed a bit more of what’s happening (Clinton - carer).

b) Role and integration of primary care post-BMT

Participants had mixed views about the involvement of community-based professionals, including GPs and community-based Nurse Practitioners, in their post-BMT care. Several participants felt that GPs had a limited role in long-term BMT follow-up care, viewing their responsibilities as principally related to general check-ups, vaccinations, prescriptions and providing referrals. Several participants also expressed the opinion that GPs may not possess the skills or knowledge necessary to provide more expert care for BMT recipients.

I’m very happy with my GP but as far as the transplant goes, he’s really just managed my inoculations. All my other general health issues. I mean, [he’s] very quick to refer me on if he can’t handle it (Trevor - BMT survivor).

My GP is very cautious. He would get me to go and have blood tests from time to time.. there are some markers that I’ve had from day one for the last 10/15 years that have been elevated. And the haematologist goes “They’re not of concern,” where the GP goes, “Well, they could be of concern.” You know what I mean? It’s only because he’s not trained or specialised … he’s a genius. The GP does a great job, and he fills a position but that’s it (Ethan - BMT survivor).

A GP for me, as good as they are, he’s for the more routine illnesses of life. Not serious conditions, anything life-threatening (Steve - BMT survivor).

I’m confident that our GP understood, and got the process, and what he [BMT survivor] went through to be able to then continue to care for him and tell him the right things to look out for and such like that (Lily - carer).

c) Communication between BMT centres and primary care practitioners

Those participants who felt they had good relationships with their GPs noted how this relied upon effective communication between their specialist BMT team and their GP.

They’re working together, I reckon Doctor [BMT Doctor] sends everything. 100 percent, and he [GP] sends Doctor [BMT Doctor] what he’s done on the computer… Somehow they have a communication thing, I don’t know how they do it, but Doctor [BMT Doctor] knows and he knows exactly what’s going on with my local GP, you know what I mean? (Fred - BMT survivor)

… they share information. Because every time I go to the hospital for my visits, I obviously take referral letters so they’re continually updating the fact of who my GP is or confirming who my GP is. And then as far as I’m aware, she’s getting copied in any blood test results that I do. And, as I said, from the GP side of things, she’s aware that she needs to provide them with information regarding blood tests (Roger - BMT survivor).

And the more information you give a GP and the more that he deals with the specialist, then his knowledge base grows where he’s not specialising it, but he has an understanding of it (Ethan - BMT survivor).

I probably can’t comment on what the hospital gave our GP. But I can just say that our GP is extremely switched on, and understood what Dad was going through. So I think for someone who maybe didn’t have, if someone didn’t have a constant GP that they saw, like a regular GP, or something like that, that they weren’t too close to, I think the experience might have been different. But I was confident that my GP knew and understood what was going on, and therefore, she would be all over it. Yeah, I didn’t have any concerns (Lily - carer).

For those patients who did not have a regular GP, paperwork from the hospital or via My Health Record was the only means to communicate with the GP.

The specialist gave me the paperwork. I gave the paperwork to the GP. He’s given me the referrals, given it to the nurse and that’s the only dealing I had with the GP (Victor - BMT survivor).

Well, I don’t know if we were asked, obviously with My Health Record, but obviously that could be a question to patients, like are you happy that a summary of what treatment you’ve had goes along with you on your My Health Record, so that if you do go to another area or place, that it follows you. And people are always aware of this in case you might forget or not think to tell them or don’t think it’s related. Sometimes patients can think it’s not really related to what I’m presenting with (Campbelle - carer).

## Discussion

The impact of BMT on survivors’ long-term health and quality of life is substantial, leading to questions about the most appropriate configuration of services and models of care for this cohort [22]. Post-BMT care must meet an individual’s needs, be feasible and should be sustainable [23]. A number of models for long-term care exist, with individual centres adopting different approaches according to local capacity, case-load, characteristics of the BMT survivor population, availability of community services and support (including carer support) and the preferences of health professionals involved in their care (primary care services, allied health and specialist services) [23]. While there have been studies describing the delivery of post-BMT care, these are mostly from the perspective of health professionals [24, 25], focusing on the availability of human and material resources [22, 25, 26] or exploring health outcomes relating to specific long-term issues [24, 27]. Dyer, Gilroy’s [10] quantitative study is an exception to this, exploring survivors’ preferences for receiving long-term care. Findings from this study revealed that most survivors preferred care to be either provided by their tertiary centre or by a satellite centre linked with or administered by the tertiary centre, with or without the option to use telehealth for consultations.

Our qualitative study builds on the findings of Dyer and Gilroy [10] and provides new insights into how variables such as relationships and experiences with specific health professionals impact the preferences of BMT survivors and carers for long-term care. Trust and respect for haematologists and other key health professionals, such as transplant co-ordinators and nurses, were important for many participants. This is unsurprising, as BMT recipients entrust their lives to specialised BMT teams. Such was the importance of this therapeutic relationship that many survivors emphasised maintaining this relationship, even where other health professionals were available at more convenient times or locations. In our study, the concept of trust indicated survivors feel empowered by their relationship with their BMT specialist, their trust in the specialist’s expertise and their willingness to do what is best for the survivor, rather than survivors experiencing a paternalistic relationship which is often associated with the concept of trust [28]. This sense of empowerment may be central to developing acceptance by survivors when transitioning their long-term care to other health professionals away from tertiary centres.

Several participants in this study emphasised the value they placed on easy access to experienced haematology nurses for advice, clarification of care and support. This finding suggests that there may be value in developing nurse-led services in all tertiary centres and in satellite centres providing BMT long-term care, such as those commonly used in international BMT and cancer centres [10] providing long-term follow-up. Further research is warranted to explore the relative merits of nurse-led post-BMT services in the Australian context, given that few such services currently exist, and expansion of long-term care will be needed as transplant activity increases and an increasing number of recipients survive long-term.

This study also raises important questions regarding the role and integration of primary care in post-BMT management. While there are good practical reasons for the involvement of GPs and community nurses in post-BMT care, many participants expressed some reticence to have their long-term care ‘handed over’ completely to their local GP, due to concerns about the specialist nature of their condition and/or the lack of a close relationship with their GP and/or the challenge associated with accessing medical care in the community. These concerns are particularly salient given the challenges currently facing GP care in Australia [29], particularly in rural and remote areas where there are chronic GP shortages, regular turnover of GPs [30], inconsistent availability [31] and knowledge gaps relating to BMT management [27]. Participants in this study also described how successful integration of GPs in the care of survivors relied upon effective communication between the tertiary centres and GPs to ensure care is based on the individual’s history and its risk factors for long-term complications [27]. Future models of care need to develop and test communication strategies and information technology solutions to continue to develop communication between hospital and primary care settings.

Finally, our study highlights the impact that contextual factors may have on the success and acceptability of post-BMT care [32]. The time and costs associated with regular travel to tertiary centres were a major issue for many BMT survivors, having a profound impact upon their quality of life and that of their cares. While increasing capacity for the provision of local care may seem the most logical response to this challenge, it raises considerable logistic difficulties. Telehealth may clearly provide one means for enabling more equitable post-BMT care and improving communication between tertiary and local health professionals. Unlike some other studies [10, 33], participants in this study were receptive to the use of telehealth. Advances in the use and acceptance of telehealth and videoconference consultations [34–37] and also the increasing use of patient monitoring via wearable devices across many areas of patient care and chronic disease management [38] provide additional tools to ensure BMT survivors achieve optimal outcomes and quality of life.

This study has some notable limitations to consider. Firstly, while the sample size allowed for data saturation, the sample was drawn from two local health districts in NSW and, therefore, may not be representative of all BMT survivors. Secondly, the study was conducted in a well-resourced area with access to BMT centres. Perspectives of BMT survivors living in more remote regions with limited access to specialist services in their districts may differ substantially. Thirdly, most participants (73%) were within 5 years post-BMT, with over half less than 2 years out, so those participants were relatively early in the long-term follow-up process. Approximately two-thirds of the participants were male which is broadly consistent with gender differences that exist in the provision of BMT in Australia [4]. We also did not collect data on ethnicity, nor explicitly explore cultural and ethnic variations in care so we are unable to comment about this. Longer-term BMT survivors may have different experiences, needs and attitudes regarding transitions in follow-up care that warrant further investigation. Despite these limitations, the findings provide important insights into the perspectives of BMT survivors and carers during the critical transition period from acute treatment completion to long-term survivorship care. They also provide important starting points for investigations into the areas not studied such as the experience of people from culturally, ethnically and linguistically diverse communities.

## Conclusion

This study highlights the importance BMT survivors and carers place on maintaining their relationships with and ongoing access to specialised BMT teams for long-term survivorship care. While some are open to receiving local follow-up, concerns exist around the function and capacity of primary care providers. As more BMT are performed, and as BMT survivors live longer post-transplant, their care needs to transition towards more sustainable shared-care models involving BMT centres and primary/community providers. However, this shift requires improved support, communication, and co-ordination across sectors. Strategies like provider education on BMT survivorship needs, optimising telehealth and other technology and clearly delineating roles/responsibilities are vital for facilitating this transition.

Ultimately, flexible individualised long-term follow-up models providing continuous access to specialised BMT expertise that leverage local care provision could improve survivor experiences, adherence to long-term follow-up guidelines and overall outcomes for this growing population.

## Data Availability

The data that support the findings of this study are available from the corresponding author upon reasonable request.
